# Impact of an Electronic Health Record-Integrated Personal Health Record on Patient Participation in Health Care: Development and Randomized Controlled Trial of MyHealthKeeper

**DOI:** 10.2196/jmir.8867

**Published:** 2017-12-07

**Authors:** Borim Ryu, Nari Kim, Eunyoung Heo, Sooyoung Yoo, Keehyuck Lee, Hee Hwang, Jeong-Whun Kim, Yoojung Kim, Joongseek Lee, Se Young Jung

**Affiliations:** ^1^ Office of eHealth Research and Business Seoul National University Bundang Hospital Seongnam Republic Of Korea; ^2^ Department of Family Medicine Seoul National University Bundang Hospital Seongnam Republic Of Korea; ^3^ Department of Pediatrics Seoul National University Bundang Hospital Seongnam Republic Of Korea; ^4^ Department of Otorhinolaryngology–Head and Neck Surgery Seoul National University Bundang Hospital Seongnam Republic Of Korea; ^5^ Department of Transdisciplinary Studies Graduate School of Convergence Science and Technology Seoul National University Suwon Republic Of Korea

**Keywords:** health records, personal, lifelog data, lifestyle management, clinical intervention, health care service, electronic health records, mobile health, telemedicine, clinical trial

## Abstract

**Background:**

Personal health record (PHR)–based health care management systems can improve patient engagement and data-driven medical diagnosis in a clinical setting.

**Objective:**

The purpose of this study was (1) to demonstrate the development of an electronic health record (EHR)–tethered PHR app named MyHealthKeeper, which can retrieve data from a wearable device and deliver these data to a hospital EHR system, and (2) to study the effectiveness of a PHR data-driven clinical intervention with clinical trial results.

**Methods:**

To improve the conventional EHR-tethered PHR, we ascertained clinicians’ unmet needs regarding PHR functionality and the data frequently used in the field through a cocreation workshop. We incorporated the requirements into the system design and architecture of the MyHealthKeeper PHR module. We constructed the app and validated the effectiveness of the PHR module by conducting a 4-week clinical trial. We used a commercially available activity tracker (Misfit) to collect individual physical activity data, and developed the MyHealthKeeper mobile phone app to record participants’ patterns of daily food intake and activity logs. We randomly assigned 80 participants to either the PHR-based intervention group (n=51) or the control group (n=29). All of the study participants completed a paper-based survey, a laboratory test, a physical examination, and an opinion interview. During the 4-week study period, we collected health-related mobile data, and study participants visited the outpatient clinic twice and received PHR-based clinical diagnosis and recommendations.

**Results:**

A total of 68 participants (44 in the intervention group and 24 in the control group) completed the study. The PHR intervention group showed significantly higher weight loss than the control group (mean 1.4 kg, 95% CI 0.9-1.9; *P*<.001) at the final week (week 4). In addition, triglyceride levels were significantly lower by the end of the study period (mean 2.59 mmol/L, 95% CI 17.6-75.8; *P*=.002).

**Conclusions:**

We developed an innovative EHR-tethered PHR system that allowed clinicians and patients to share lifelog data. This study shows the effectiveness of a patient-managed and clinician-guided health tracker system and its potential to improve patient clinical profiles.

**Trial Registration:**

ClinicalTrials.gov NCT03200119; https://clinicaltrials.gov/ct2/show/NCT03200119 (Archived by WebCite at http://www.webcitation.org/6v01HaCdd)

## Introduction

The Precision Medicine Initiative (PMI) is a nationwide project in the United States that aims to build a longitudinal cohort representative of the American population by collecting samples and data from 1 million participants [1,2]. Precision medicine focuses on identifying approaches that will be effective for patients, based on genetic, environmental, and lifestyle factors. In 2016, US $130 million was allocated to the US National Institutes of Health to build a national, large-scale research participant group (cohort), and US $70 million was allocated to the US National Cancer Institute to lead efforts in cancer genomics as part of the PMI for Oncology [1,3]. The PMI was launched as part of this initiative, and the All of Us Research Program [4] is a key element of the PMI project. This research program aims to collect the biological, environmental, and behavioral data generated by each participant to gain better insights into individualized care perspectives.

Lifelog patient-generated health data, considered important for PMI implementation, form the next frontier in patient engagement and customized health care [5,6]. As indicated by the name, patient-generated health data require the participation of the patient. Through the availability of numerous devices, and mobile apps compatible with these devices, patients can collect their own health-related lifestyle data, which can be aggregated with their clinical data into their personal health file. This aggregate of lifestyle and clinical data stored in the personal health file is termed lifelog data.

Unfortunately, it is very difficult to accumulate lifestyle data such as daily dietary intake, sleep log, and stress in a longitudinal record. Several studies have been conducted in health care using data from the personal health record (PHR) and its related devices to verify its usability and feasibility [7-12]. Health care apps that are compatible with mobile devices and can collect personal health data serve as tools to improve patient adherence to self-management in a variety of diseases. However, they have limited utility for long-term use, owing to the additional patient burden created by lack of expertise and the absence of professional oversight for evaluation of progress [12-17]. Therefore, a significant need for an intermediate model has been emerging, in which patients and medical staff communicate with each other, ultimately increasing patient adherence for health promotion purposes.

In a previous study [18], we showed that patients with chronic diseases are more likely to use a PHR system that is integrated into a comprehensive electronic health record (EHR). In addition, we showed that patients with a higher number of chronic diseases tend to use PHRs more actively, employing the self-administered function. Our study was, to our knowledge, the first to determine factors affecting adherence to, and use of, a self-administered function of PHR tethered to a comprehensive EHR, which is an important determinant of active use of a PHR.

This study primarily aimed to determine whether patients would use a PHR app to record lifelog data and to ascertain the ease with which these data could be shared with clinicians during appointments. In addition, we sought to understand whether patients would use the PHR actively and voluntarily, and whether these data could improve clinical profiles. This study therefore aimed to demonstrate (1) the development of an EHR-tethered PHR system that can retrieve data from a wearable device, and (2) the efficacy of such a system paired with a lifelog data-driven intervention modality. In particular, as a further development of our previous studies, we constructed an EHR-tethered PHR module in an EHR-friendly hospital (where a comprehensive EHR system has been operated successfully for over 12 years) and designed a PHR interface based on the lifestyle data requirements of physicians in a clinical setting.

## Methods

### MyHealthKeeper: Improvement on Conventional Electronic Health Record-Tethered Personal Health Records

As the first hospital to attain Healthcare Information and Management Systems Society stage 7 status outside of North America, Seoul National University Bundang Hospital (SNUBH; Seongnam, Republic of Korea) introduced a comprehensive EHR system in all the divisions of the hospital in 2003, launching a related PHR service in 2013 [19]. The hospital has 1340 beds and over 5000 daily outpatients. A task force team was established to conduct a needs analysis and develop the MyHealthKeeper PHR module in this study site. To understand clinicians’ unmet needs corresponding to PHR data, and to delineate the most important functionality to be incorporated into a PHR module, we employed a cocreation process.

**Figure 1:**
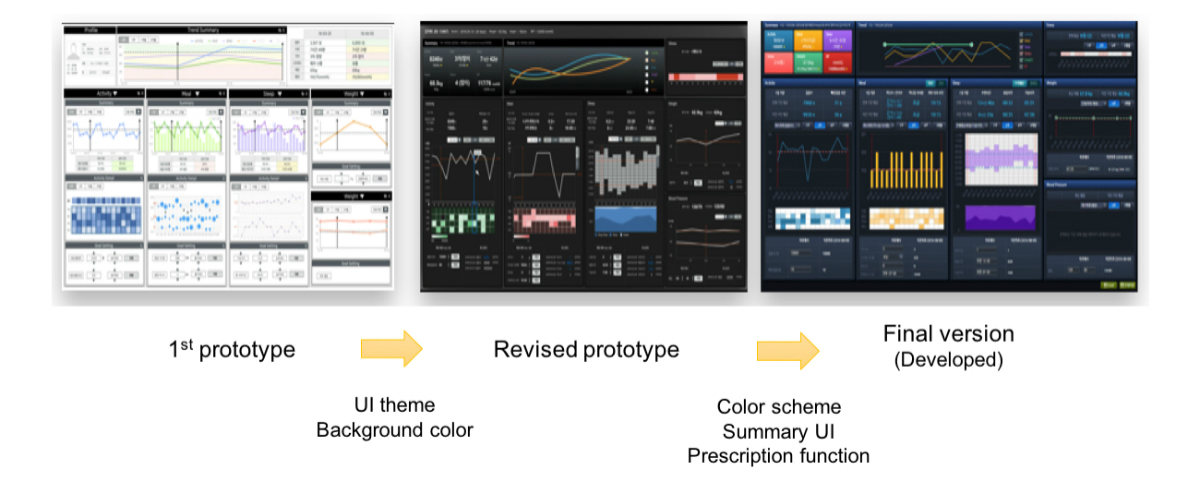
Developmental process of the electronic health record-tethered personal health record system. UI: user interface.

Cocreation is an alternative, collaborative, user experience-based research approach that increases the user’s direct involvement [20,21]. In practice, this often takes the form of a collaborative workshop in which business stakeholders, researchers, designers, and end users explore a problem and generate solutions together, considering their different approaches, needs, and points of view. Thus, we conducted a 1-day collaborative workshop for the cocreation process to understand comprehensive PHR data requirements of clinicians, such as practical visualization factors that can reflect patient lifestyle and health behavior. A total of 15 researchers participated in the workshop: 2 clinicians, 4 informatics specialists, 3 developers, and 6 user experience specialists. Figure 1 shows the developmental process of our PHR module interface.

Participants were divided into 3 teams to share and analyze opinions regarding the PHR interface and design, organized according to the following questions: (1) What lifestyle-related data categories need to be displayed? (2) What is an effective visualization summary for the clinician? (3) How can we effectively deliver personal feedback in the form of a clinical prescription?

**Figure 2:**
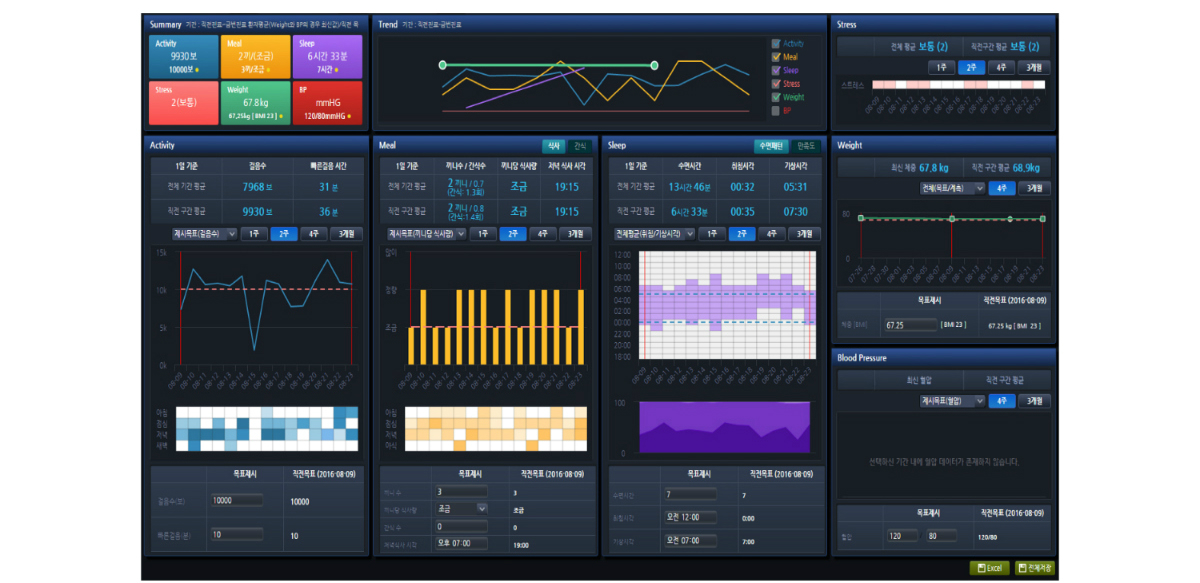
MyHealthKeeper interface design.

**Figure 3:**
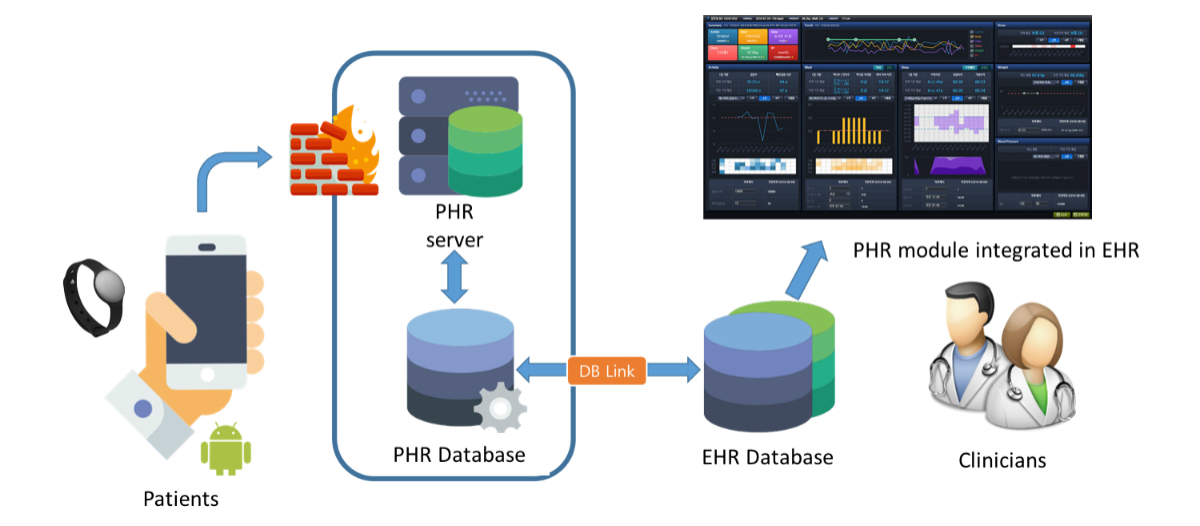
Personal health record (PHR) data flow overview. DB: database; EHR: electronic health record.

We used the answers to these questions to guide the creation of a final interface design prototype through idea clustering, screen sketching, continuous analysis, and improvement. Figure 2 presents the clinical PHR interface screen that we created on the basis of this end-user needs analysis. From the data flow point of view, the mobile app data and activity tracking device log generated by each patient every day were stored on the PHR server. Data were transferred and saved with an independent PHR identifier for data protection, and the data for each patient were consolidated by a PHR database and conventional EHR database link by matching the original patient identifier. Clinicians could review these data on the PHR module interface and provide health-related lifestyle-management feedback on individual activity, sleep, meal consumption, or stress status. Figure 3 demonstrates the data flow in this study.

The MyHealthKeeper interface design showed patient-generated lifestyle data in a graphical format on a clinician’s EHR screen. Physical activity was reflected by step count and was visualized as a line graph with the day plotted on the x-axis and the total daily step count value plotted on the y-axis. A heat map was also displayed, with 4 rows depicting data pertaining to the morning, afternoon, evening, and nighttime, and columns pertaining to each day, as in the line graph. We set the default prescription value of daily activity step count to 10,000. We constructed the patient diet records and sleep log plots in the same manner as the activity plots. To visualize the sleep log, we chose a stacked area graph format rather than a heat map, as per clinicians’ requirements. Weight and blood pressure change values were plotted as line graphs. Daily stress was summarized as a form of heat map graph. Each lifestyle-related health data plot contained a prescription section for the clinician, which allowed the clinician to specify a healthier daily routine for the patient, considering goals such as preventing weight gain, increasing physical movement, or increasing or decreasing the sleep period.

### MyHealthKeeper: Mobile App

We developed an Android operating system-based mobile phone app designed to collect health-related lifestyle data and tested it in an experimental patient group. The objective of using personal feedback coaching based on a mobile app and a wearable activity tracker is to lose weight for a healthy lifestyle. We designed an app compatible with a commercially available wearable activity device (Misfit Shine; Misfit Wearables Corporation, Burlingame, CA, USA), to collect daily activity data automatically [22].

**Figure 4:**
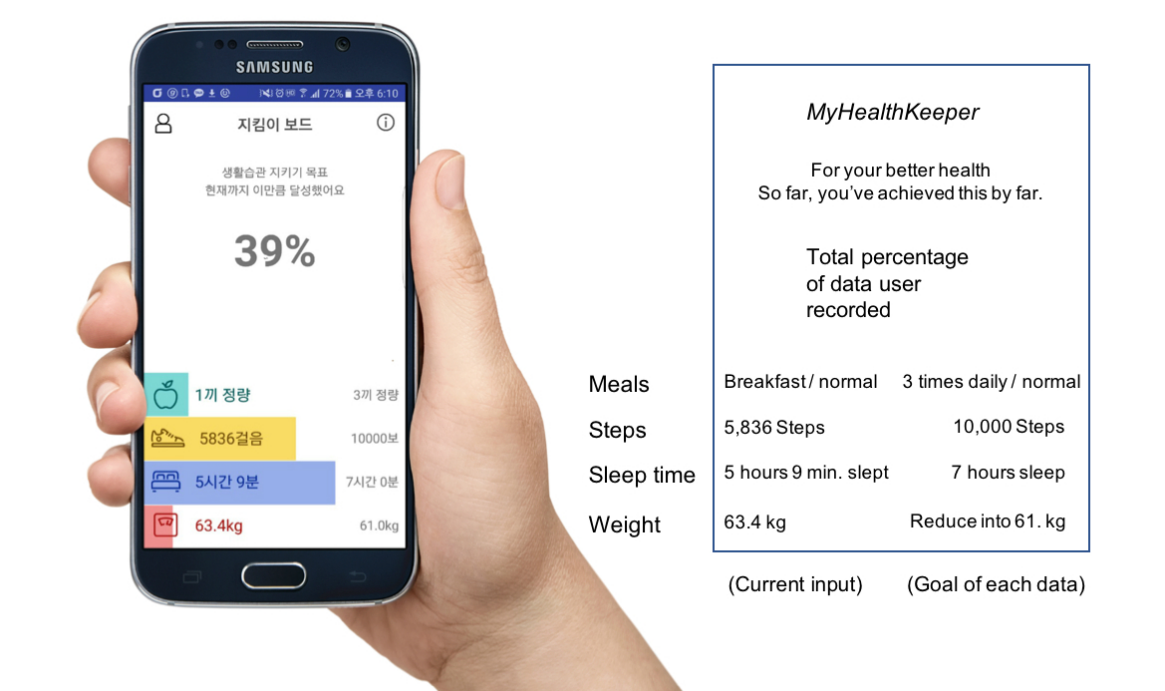
MyHealthKeeper mobile app. Left: Korean version interface; right: English-translated description.

The MyHealthKeeper app was composed of several logging pages named as follows: daily meal, physical exercise activity, sleep log, stress, blood pressure, and weight value. The main page of this app (Figure 4) showed the total amount of collected data at the top of the interface (eg, “39%”), current input data at the left side, and the goal of each lifestyle-related status at the right side. At any time, the intervention group participants could check their activity and dietary status with a mobile phone, and access food intake allowances remaining for the day and the amount of moderate- to vigorous-intensity physical activity needed to reach the daily goal. (See Multimedia Appendix 1 for more app pages.)

### Clinical Study Design and Participant Recruitment

**Figure 5:**
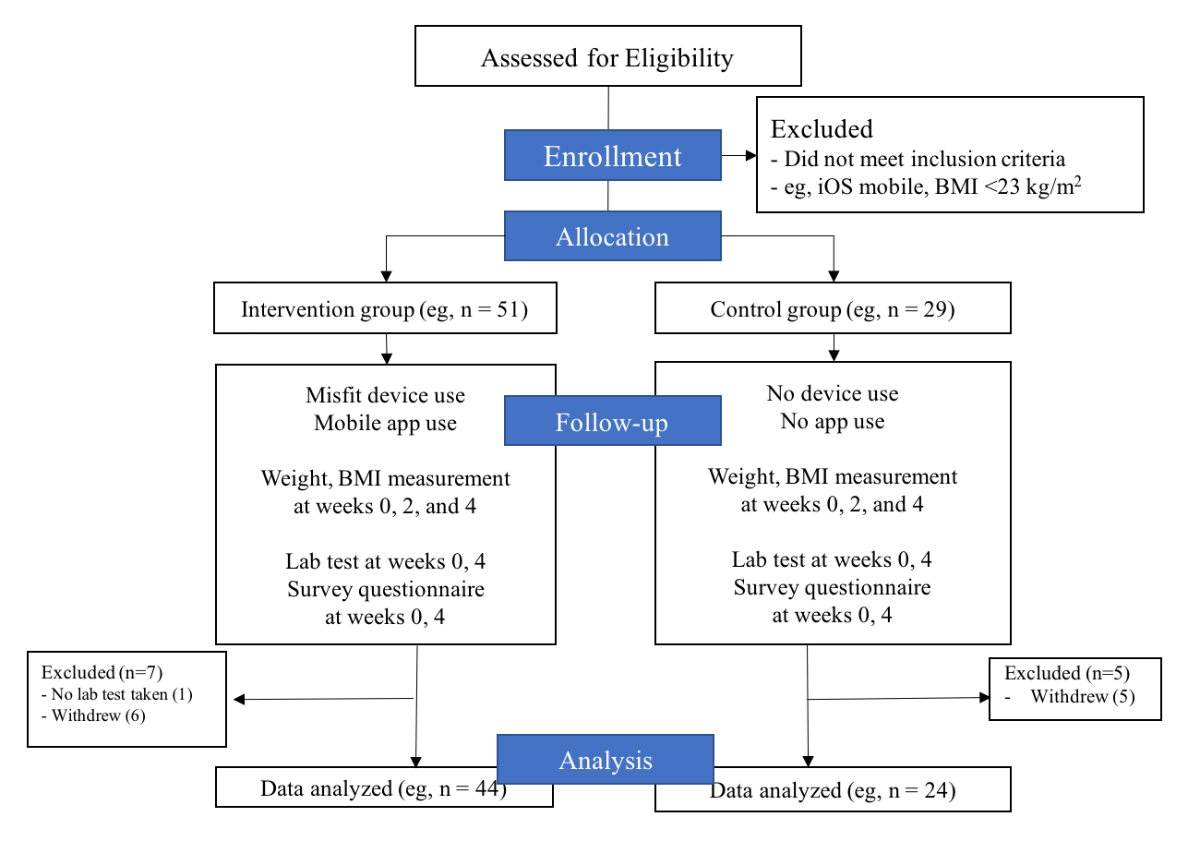
Clinical trial study design.

Our whole study period was 8 months. System planning and the interface design workshop for gathering clinicians’ opinions required 2 months. Implementation took 3 months, and after launching the system, the clinical trial was performed for 4 weeks. We conducted a prospective randomized clinical trial in 80 patients who visited the SNUBH outpatient clinic between the months of July and September 2016. We set the following inclusion criteria for enrollment in the trial: (1) patients who provided prior consent to complying with self-management, (2) patients without cardiopulmonary disease, cancer, or other acute diseases, and (3) patients with a body mass index (BMI) of over 23 kg/m^2^. Figure 5 describes the overall clinical trial study design.

We excluded patients who would not be able to use a mobile app and a wearable device and those who were pregnant. We obtained written informed consent from all participants. All study participants completed a paper-based survey, a laboratory blood test, a physical examination, and an opinion interview.

This study was approved by the SNUBH Institutional Review Board (B-1504-296-302), which was also registered with ClinicalTrails.gov (registration number: NCT03200119), and this study is reported in accordance with the Consolidated Standards of Reporting Trials (CONSORT)-EHEALTH checklist (Multimedia Appendix 2) [23].

**Figure 6:**
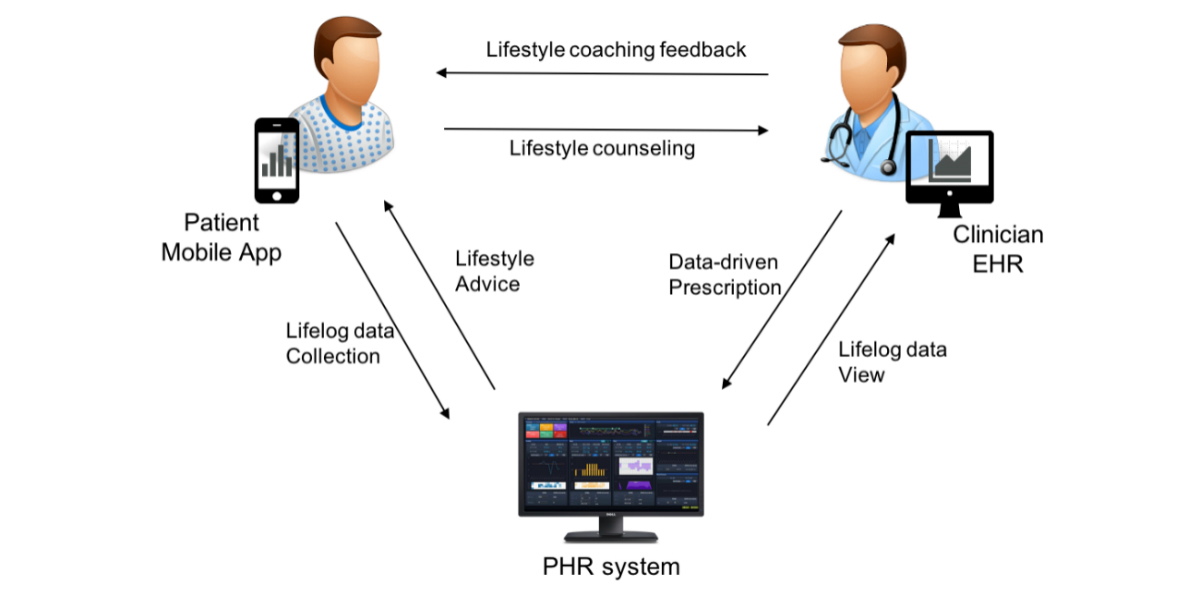
Patient-clinician-system workflow. EHR: electronic health record; PHR: personal health record.

### Personal Health Record-Based Interventions

The main objective of this clinical trial was to analyze the effectiveness of personalized health care management by mobile app use and clinical coaching feedback with an EHR-integrated PHR module. To this end, we randomly assigned enrollees to 2 groups: a PHR-based mobile app and clinical intervention group, and a conventional treatment control group.

The first group (PHR intervention group) received the wearable device, the mobile app software, personal coaching, and the intervention based on the PHR module to encourage a healthy lifestyle. The participants were educated about lifestyle modifications needed to lose weight and were trained in the use of the Android operating system-based mobile phone app (designed to collect lifestyle data); Figure 6 describes this workflow.

Individualized goals for diet and physical activity were prescribed for each participant by the clinician during a biweekly outpatient visit. The patient lifestyle data were displayed as a summary plot on the EHR interface accessed at the participating hospital and were reviewed by the clinician. Further lifestyle modification was encouraged and prescribed on the basis of the EHR-integrated PHR module. Clinicians made at least one comment for patients whenever they visited the outpatient clinic; therefore, a total of 88 feedback comments (2 visits per patient) or lifestyle health prescriptions were issued on the PHR module.

For example, a 36-year-old male patient visited our outpatient clinic. He was found to be prediabetic in his regular health checkup. He was obese (height 170 cm, weight 74 kg) and had a sedentary lifestyle because he was an office worker. His lipid profile was not good, with a triglyceride level of 13.6 mmol/L, indicating that he usually ate a lot of carbohydrates. At the first visit, his doctor found his daily step counts were below 4000 steps and he ate a lot between meals. Therefore, the doctor made the following written lifestyle health prescription: “Please cut down on every snack between meals and walk more than 10,000 steps every day.” Furthermore, the clinician set a lifestyle goal of a weight loss of at least 500 grams below his current weight by the next clinic visit (2 weeks later). The patient followed the doctor’s recommendations because he noticed that he could share his lifelog data with his doctor via the EHR-tethered PHR app. In fact, he had lost 600 grams by the next visit, and all the lifelog data were naturally integrated into his EHR via the PHR app.

The control group of patients did not receive the lifestyle modification app or the wearable device. They received conventional care pertaining to lifestyle modification for achieving weight loss goals during the 4-week study period.

### Clinical Study Outcome Measure

The primary outcome measure of this clinical trial was weight change. Body weights before and after the PHR-based clinical intervention were recorded and analyzed. We defined BMI as the body mass divided by the square of the body height, expressed in units of kg/m^2^, and we analyzed the difference in BMI before and after the study at the end of the study period. We analyzed the secondary outcomes of the study—changes in blood biochemical parameters (cholesterol, triglycerides, high-density lipoprotein cholesterol, and low-density lipoprotein cholesterol)—for each participant. Any decrease in body weight during the study period (4 weeks) was defined as successful weight reduction. It is very important that the measurement be taken using the same method and in the same conditions to ensure uniformity between participants and in the same participant over time. In our study, a skilled nurse helped to measure the patient’s body weight in the hospital health checkup center with the conventional health checkup process (place, dress). Fasting body weight was measured for laboratory checkup.

### Statistical Analysis

Results are presented as mean (SD). We analyzed differences in various parameters between the PHR-based intervention group and the control group using the chi-square test as appropriate. We used a paired *t* test to examine changes in primary or secondary outcomes in the 2 groups. Statistical analyses were performed using IBM SPSS version 18.0 (IBM Corporation), and *P*<.05 was considered statistically significant.

## Results

We randomly assigned 80 participants to either the PHR-based intervention group (n=51) or the control group (n=29). After exclusions and withdrawals (Figure 5), 68 participants completed the study.

### Validation of the Personal Health Record System: Analysis of the Clinical Trial

Among the 68 enrolled patients who completed this study, 44 patients were assigned to the PHR intervention group and the rest (24 patients) were assigned to the control group receiving conventional care. Table 1 and Table 2 show the demographic and baseline characteristics of study participants.

**Figure 7:**
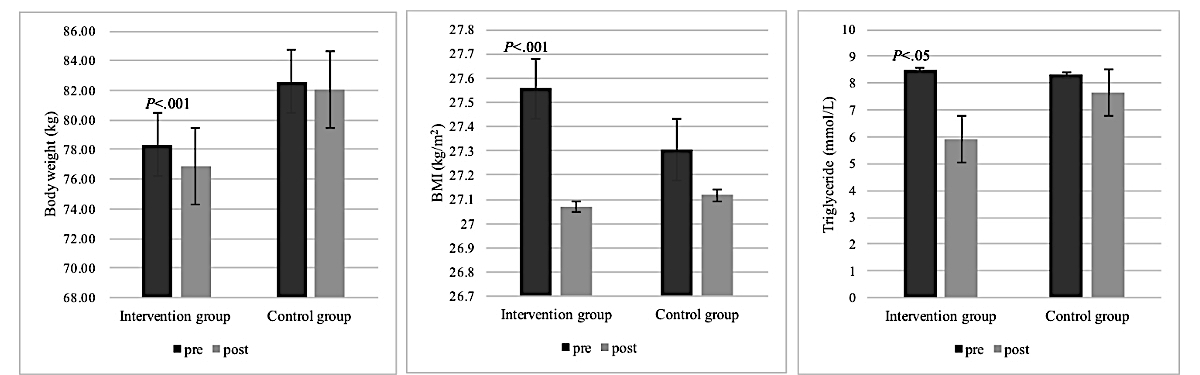
Changes in weight, body mass index (BMI), and triglycerides in the 2 groups before (pre) and after (post) the intervention. Error bars indicate 95% CI.

Our clinical trial study results revealed a significant change in participants’ clinical profiles after using the PHR-based clinical intervention as guided by the physician. The PHR intervention group participants who used the MyHealthKeeper mobile app every day and received lifestyle feedback counseling from the clinician showed significantly larger changes in weight, BMI, and triglyceride values than those in the control group (Table 3). The PHR intervention group lost significantly more weight than the control group (mean 1.4 kg, 95% CI 0.9-1.9; *P*<.001; Figure 7). Figure 7 depicts changes in biochemical parameters such as BMI (mean 0.4 kg/m^2^, 95% CI 0.3-0.6; *P*=.000) and triglyceride (mean 2.6 mmol/L, 95% CI 17.6-75.8; *P*=.002) in the 2 groups during the study period.

**Table 1:** Demographic data of study participants (n=68).

Characteristics		Intervention group (n=44)	Control group (n=24)	*P* value
Age (years), mean (SD^a^)		37.5 (8.7)	41.3 (11.2)	.30
**Sex, n (%)**				.68
	Male	30 (68)	22 (92)	
	Female	14 (32)	2 (8)	
**Education level, n (%)**				.64
	High school degree	6 (14)	4 (17)	
	College degree	32 (74)	15 (63)	
	Master’s or doctorate	5 (11)	5 (21)	
**Occupation, n (%)**				.13
	Professional	10 (23)	7 (30)	
	Office worker	15 (63)	10 (42)	
	Self-employed	5 (11)	2 (8)	
	Manufacturing or services	4 (9)	3 (13)	
	Unemployed	10 (23)	1 (4)	
**Living status, n (%)**				.60
	Living with someone	37 (84)	23 (96)	
	Living alone	7 (16)	1 (4)	
**Marital status, n (%)**				.30
	Single	11 (25)	2 (8)	
	Married	33 (75)	22 (92)	

^a^SD: standard deviation.

**Table 2:** Baseline clinical profiles of study participants.

Characteristics	Intervention group (n=44)	Control group (n=24)	*P* value
	Mean (SD^a^)	Mean (SD)	
Weight (kg)	78.3 (11.8)	82.6 (8.4)	.13
Height (cm)	168.0 (8.7)	174.0 (8.0)	.01
BMI^b^ (kg/m^2^)	27.6 (3.0)	27.3 (2.4)	.72
Cholesterol (mmol/L)	10.5 (1.8)	11.2 (1.9)	.12
HDL^c^ cholesterol (mmol/L)	2.8 (0.5)	2.8 (0.5)	.84
LDL^d^ cholesterol (mmol/L)	6.2 (1.3)	6.8 (1.5)	.07
Triglyceride (mmol/L)	8.5 (6.5)	8.2 (3.8)	.90

^a^SD: standard deviation.

^b^BMI: body mass index.

^c^HDL: high-density lipoprotein.

^d^LDL: low-density lipoprotein.

**Table 3:** Clinical profile changes in participants in the intervention (n=44) and control (n=24) groups.

Characteristics		Prestudy value	Poststudy value	*P* value
		Mean (SD^a^)	Mean (SD)	
**Weight (kg)**				
	Intervention group	78.3 (11.9)	76.9 (11.2)	<.001
	Control group	82.5 (8.41)	82.0 (8.3)	<.05
**BMI^b^ (kg/m^2^)**				
	Intervention group	27.6 (3.0)	27.1 (2.8)	<.001
	Control group	27.2 (2.4)	27.1 (2.4)	.07
**Cholesterol (mmol/L)**				
	Intervention group	10.5 (1.8)	10.4 (1.7)	.61
	Control group	11.2 (1.9)	11.3 (2.2)	.79
**HDL^c^cholesterol (mmol/L)**				
	Intervention group	2.8 (0.5)	2.9 (0.5)	.20
	Control group	2.8 (0.7)	2.8 (0.5)	.59
**LDL^d^cholesterol (mmol/L)**				
	Intervention group	6.2 (1.3)	6.3 (1.4)	.67
	Control group	6.8 (1.5)	6.9 (1.6)	.92
**Triglyceride (mmol/L)**				
	Intervention group	8.5 (6.5)	5.9 (3.0)	<.05
	Control group	8.3 (3.8)	7.6 (3.8)	.35

^a^SD: standard deviation.

^b^BMI: body mass index.

^c^HDL: high-density lipoprotein.

^d^LDL: low-density lipoprotein.

## Discussion

In this study, we constructed an EHR-tethered PHR module named MyHealthKeeper and implemented this software in an EHR-friendly hospital, with a 12-year experience in EHR use. Lifelog patient-generated health data, considered important for PMI implementation, require the participation of the patient. We gathered this patient-generated, lifestyle-related health information with a mobile app and an activity tracking device, and transferred the information to the PHR data server to make a summary view based on the practical needs of the clinicians. These requirements were incorporated into the MyHealthKeeper system design. Moreover, to validate the effectiveness of the system, we performed a 4-week clinical trial. The result of the trial showed that PHR use correlated significantly with larger changes in body weight and clinical parameters, signifying a better health status than with conventional treatment.

### Comparison With Prior Work

We aimed to demonstrate the development of an EHR-tethered PHR system that can retrieve data from a wearable device, in conjunction with a lifelog data-driven intervention. A previous study reported that patient-generated health data can improve communication between patients and health care professionals, with a concomitant improvement in patient mental outlook [24]. This study showed that patients actively used the PHR system to improve the doctor-patient relationship. However, PHR systems have pros and cons in the real clinical setting and from continuous development and use. In addition, there was a study to identify factors influencing the willingness of health care consumers to use PHRs in Korea [25]. Only a few studies have been conducted with patients using the self-administered features of EHR-tethered PHR systems, which can enable shared health care and patient-centered practice. A previous study examined the usability of an EHR-integrated PHR system tied in with patient clinical records, which functionally focused on finding an appointment time, reviewing test results, and managing medication dosages [26]. This study was performed as a Web-based patient portal use survey, including video-recorded poststudy interviews for health management purposes, with a patient-centric viewpoint. Taha et al [11] concluded that participants’ perceptions of the PHR system were positive, and patients were very receptive to the idea of using PHR systems to help them perform health management tasks. While their study was highly patient centric, our study focused on both patient and clinician experiences, with a fully integrated PHR module. Furthermore, we studied the impact of PHR-based clinical interventions on clinical profile changes in participants. Mishuris et al [27] also studied PHR and EHR integration usability for clinical workflow design. They performed a qualitative study using two rounds of semistructured interviews with primary care providers, health information software developers, and health care researchers. This study suggested a framework for how to integrate external data into provider workflow in an efficient and effective way. However, the researchers provided only a prototype design, not a complete implementation result.

Recently, several studies have been conducted on lifestyle intervention [8,9,13,14,28,29]. A study protocol for a pragmatic randomized controlled trial for physical activity coaching in patients with chronic obstructive pulmonary disorder, including management of patient-centered daily activity, tracking of cardiovascular disease risk factors, and monitoring of quality of life measures, was published. A different study used EHR data to evaluate a physician-developed lifestyle plan for obese patients in primary care [10,28]. Simple lifestyle changes and dietary interventions were suggested in the plan, which was distributed to obese patients by a family physician as part of routine clinical care. This study reported significant weight loss in older men and a significant reduction in systolic blood pressure in all participants. Although the lifestyle coaching intervention concept was similar to that used in our study, this study did not use a personal lifelog or wearable devices.

### Strengths of the Study

In our previous study [18], we found, to our knowledge, for the first time that patients with chronic diseases tended to use PHR more actively, particularly the self-administered function. According to this previous study, as a first step to move from rudimentary stand-alone PHRs to integrated PHRs and exploratory research, we reported that data gathered from EHR-tethered PHRs may be used to improve PHRs by implementing patient-centric features on the system. Through a cocreation workshop, we obtained detailed requirements from clinicians, to guide our PHR system design. We were thus able to improve on conventional EHRs and incorporate functionality frequently used in a clinical setting. A strong key point of this study is that the clinical trial was conducted to validate the effectiveness of our PHR system, MyHealthKeeper, during a 4-week period, with a wearable activity tracker to collect individual physical activity data.

### Limitations and Conclusions

Owing to practical constraints, this study could not provide a longitudinal observation of the EHR-tethered PHR system. Because of the short clinical trial period, it was difficult to determine a causal relationship, and the study did not provide information about the precise improvement in the health outcomes of PHR users. However, on the basis of this study protocol, we hope to derive and apply many PHR features of an EHR-tethered PHR system in further studies. Although the clinical trial period was short, a major difference between our study and the others is that we developed an integrated PHR system into an ordinary EHR system. As a preliminary observation, the primary objective of our study was to demonstrate the development of an EHR-integrated PHR system for clinicians to help patients make lifestyle changes and to determine whether patients followed their doctors’ recommendations that are shared via the PHR app. Moreover, the sample number in the clinical trial was small (only a few clinicians were included). Nevertheless, because this study was conducted in a tertiary care general university hospital where an EHR has been in place for 12 years, we hope to implement this system throughout the facility, including a larger number of patients and clinicians in the future. With this integrated PHR system, we also expect to further study longitudinal follow-up and continuous patient engagement.

## Appendix

Multimedia Appendix 1 Detailed mobile app pages per topic.

Multimedia Appendix 2 CONSORT-EHEALTH checklist (V 1.6.1).

## Abbreviations

BMI: body mass index
CONSORT: Consolidated Standards of Reporting Trials
EHR: electronic health record
PHR: personal health record
PMI: Precision Medicine Initiative
SNUBH: Seoul National University Bundang Hospital
