# Supplementary figures and images for: Impact of an Electronic Health Record-Integrated Personal Health Record on Patient Participation in Health Care: Development and Randomized Controlled Trial of MyHealthKeeper
Source: J Med Internet Res. 2017 Dec 7;19(12):e401. doi: 10.2196/jmir.8867 (PMC5740264; doi:10.2196/jmir.8867)

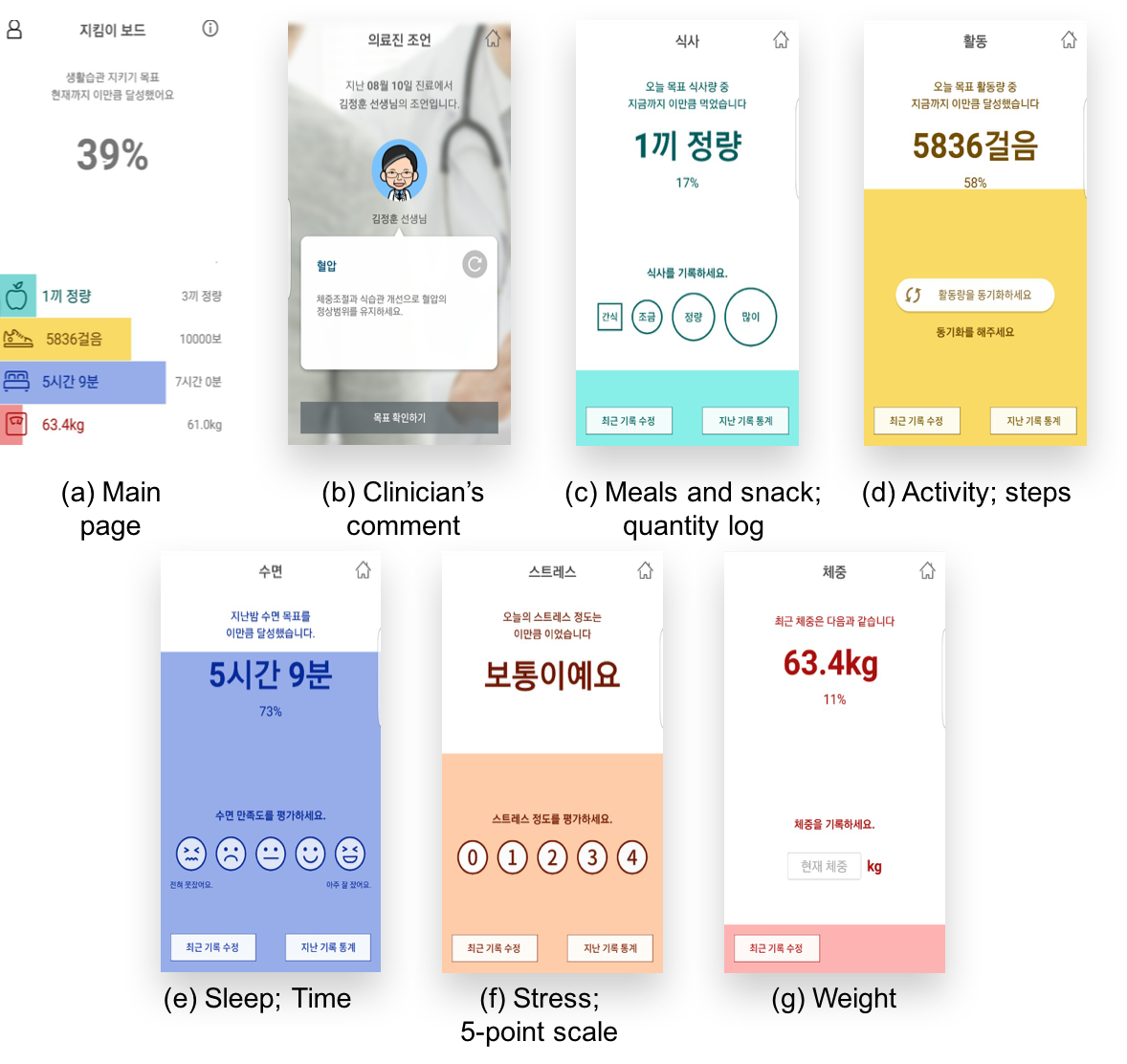

Supplement: Multimedia Appendix 1 [file jmir_v19i12e401_app1.png]
